# Design and Implementation of Broadband Hybrid 3-dB Couplers with Silicon-Based IPD Technology

**DOI:** 10.3390/mi14050932

**Published:** 2023-04-25

**Authors:** Mengmeng Xu, Jiangtao Su, Ruijin Wang, Zhongjie Lin, Weiyu Xie, Jun Liu

**Affiliations:** 1 School of Electronic Information, Hangzhou Dianzi University, Hangzhou 310018, Chinaljun77@hdu.edu.cn (J.L.); 2 Key Laboratory of Large Scale Integrated Design, Hangzhou 310018, China

**Keywords:** integrated passive devices (IPD), directional couplers, defective ground structure (DGS), wiggly lines

## Abstract

Heterogeneous integration (HI) is a rapidly developing field aimed at achieving high-density integration and miniaturization of devices for complex practical radio frequency (RF) applications. In this study, we present the design and implementation of two 3 dB directional couplers utilizing the broadside-coupling mechanism and silicon-based integrated passive device (IPD) technology. The type A coupler incorporates a defect ground structure (DGS) to enhance coupling, while type B employs wiggly-coupled lines to improve directivity. Measurement results demonstrate that type A achieves <−16.16 dB isolation and <−22.32 dB return loss with a relative bandwidth of 60.96% in the 6.5–12.2 GHz range, while type B achieves <−21.21 dB isolation and <−23.95 dB return loss in the first band at 7–13 GHz, <−22.17 dB isolation and <−19.67 dB return loss in the second band at 28–32.5 GHz, and <−12.79 dB isolation and <−17.02 dB return loss in the third band at 49.5–54.5 GHz. The proposed couplers are well suited for low cost, high performance system-on-package radio frequency front-end circuits in wireless communication systems.

## 1. Introduction

With the rapid development of wireless communication techniques in recent years, the frequency bands occupied by civilian and military communication fields have been extremely expanded, together with the rigid requirement of power, efficiency, and foot-print in the design. Traditional silicon-based integrated circuit techniques are confronting great challenges to satisfy those commands simultaneously. As a solution, heterogeneous integration (HI) has emerged as a key technique to address these challenges. By integrating silicon-based and compound circuits with different purposes, HI provides a way to meet the stringent requirements of modern wireless communication systems [1,2,3].

Integrated Passive Device (IPD) technology reflects the concept of HI, which can package passive components together with some active circuits to achieve integrated circuits with high integration, low cost, and good performance. In contrast to Low Temperature Co-fired Ceramic (LTCC) technology, IPD technology has the advantages of high processing accuracy, low cost, and small size [4,5]. This design concept has been applied to implement low noise amplifiers [6], power amplifiers [7,8], and voltage-controlled oscillators [9,10].

A variety of passive components have been designed and fabricated in the IPD technology, such as the power dividers [11], filters [12,13], and couplers [14,15,16,17,18]. Among these components, directional couplers, though possibly one of the most widely used components in microwave circuits, have had few successful attempts based on IPD technology so far. In addition to the traditional applications in power distribution and synthesis, directional couplers can also be integrated into the design of active devices such as mixers [19], power amplifiers [20], and phase shifters [21]. The coupler circuit designed based on IPD technology can obtain smaller sizes, which is conducive to the miniaturization of devices [14]. The proposed IPD couplers in [15] are verified to have wider relative bandwidths and smaller sizes in comparison with the Printed Circuit Board (PCB) process. Thanks to thick metal layers and comparable substrate resistivity with GaAs technology, the coupler of IPD technology can achieve better insertion loss [16]. However, the coupling on IPD technology is usually weak due to the lack of multilayer profiles. A recent study [18] proposed a potential solution to this issue by utilizing synthesized coplanar waveguides (CPWs) constructed with lumped elements. Nevertheless, the research on IPD technology has been limited to a few specific applications, with insufficient investigation into the development of broadband and strong coupling couplers, particularly those capable of functioning across multiple bands.

In this paper, two 3 dB directional couplers with a wide bandwidth based on silicon-based IPD technology are introduced. The type A coupler employs a DGS structure to enhance coupling, leading to improved isolation and return loss performance. The measurement results demonstrate that the type A coupler achieves a relative bandwidth of 60.96% with isolation of <−16.16 dB and return loss of <−22.32 dB over the frequency range of 6.5–12.2 GHz. Wiggly-coupled lines are utilized in the type B coupler to improve directivity in three frequency bands. The type B coupler was measured to have <−21.21 dB isolation and <−23.95 dB return loss in the first band at 7–13 GHz, <−22.17 dB isolation and <−19.67 dB return loss in the second band at 28–32.5 GHz, and <−12.79 dB isolation and <−17.02 dB return loss in the third band at 49.5–54.5 GHz. This paper is organized as follows. Section 2 presents the design methods, the optimization process, and simulation results of the two types of couplers. Section 3 describes the measurement methods, presents the measurement results, and includes a discussion with relevant literature. Section 4 concludes the paper.

## 2. Design of the Proposed Coupler

### 2.1. IPD Technology

IPD technology is proposed as a three-dimensional integration technology from the vertical to solve difficulties triggered by the termination of Moore’s Law. Compared to LTCC technology, IPD technology has finer line width and line spacing, resulting in fewer parasitic effects, which allows integrated passive devices to achieve smaller size, lower cost, and higher integration. Therefore, high-density capacitors, high-quality factor inductors, and precise resistors can be realized with high precision in the IPD process [22]. More importantly, the IPD process, as a type of Wafer Level Packaging (WLP) process, is able to package die in different processes together through vias, largely increasing the integration and flexibility of the system [23].

**Figure 1:**
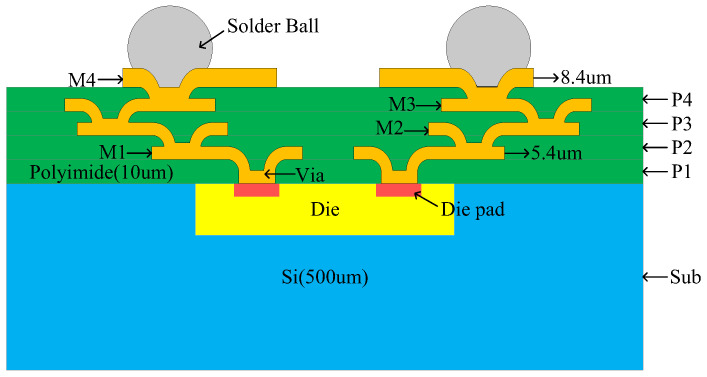
Schematic diagram of the integrated passive devices (IPD) process stack of Si substrate.

The cross-sectional view of the silicon-based IPD process used in this paper is shown in Figure 1. As can be seen, other process dies can be embedded into the silicon substrate using WLP technology and connected to the external environment through die pads, vias, and solder balls using embedded fan-out packaging. The IPD process consists of four metal layers (M1, M2, M3, and M4) made of copper (Cu) and four low dielectric constant material layers (P1, P2, P3, and P4) made of polyimide. Each dielectric layer has a thickness of 10 µm. Copper metal layers M1, M2, and M3 are embedded in P2, P3, and P4, respectively, with a thickness of 5.4 µm. The M4 metal layer is placed on top of the P4 dielectric layer, with a thickness of 8.4 µm, which results in less conduction loss in this layer. The dielectric constant ($\varepsilon_{r}$) and resistance per unit length of the silicon substrate are 11.9 and >3 kΩ·cm, respectively, and it can be thickened to 500 µm. The dielectric polyimide has a dielectric constant of 3.2 and the loss tangent of 0.009. This higher substrate resistivity, thicker substrate, and the smaller dielectric loss tangent allow for lower losses in the IPD technology. In addition, the minimum line and gap widths for all metal layers are both 15 µm. It is worth noting that the silicon-based IPD process used in this paper has better reliability, stability, and lower cost compared to the glass-based IPD process, as silicon material has better performance at high frequencies and temperatures and is cheaper. In addition, due to the thinner dielectric layer of the silicon-based IPD process, higher integration and smaller size can be achieved.

In the IPD process, since the thickness of the metal layer is comparable to the thickness of the dielectric layer, it is difficult to achieve coupling lines with high even mode impedance and low odd mode impedance. Due to the resolution limitation, the edge-coupled structures on the IPD process generally result in very weak coupling [18].

### 2.2. Type A IPD Coupler

**Figure 2:**
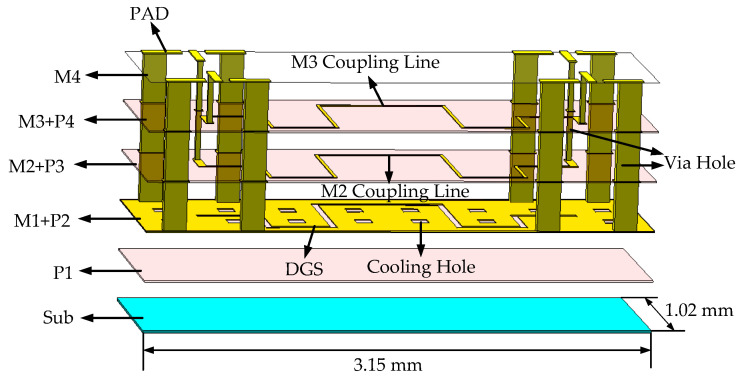
A 3D view of the type A IPD coupler.

The three-dimensional (3D) view of the proposed type A coupler is shown in Figure 2. To reduce radiation loss, the type A IPD coupler uses M2 and M3 metal layers for broadside-coupling. However, due to processing limitations, the line width of the coupling line cannot be too thin, resulting in weak coupling and low directivity when using only broadside-coupling. In order to solve these difficulties, the DGS structure is etched on the M1 grounding metal layer to enhance coupling. Via holes are utilized to connect the signals to the top layer, and the cooling holes are required for machining. Additionally, four ground–signal–ground (GSG) pads are matched to 50 Ω. To further minimize the size of the coupler, both the coupling lines and the DGS are arranged in a serpentine configuration, resulting in a final circuit size of 3.15 mm × 1.02 mm. During the initial design stage, the coupling lines were straight. A coupling line width of 20 µm was selected, and the coupling line length was determined to be 4193 µm by

(1) $$l=\frac{c}{4f\sqrt{\varepsilon_{r}}}$$

where $\varepsilon_{r}=3.2$ is dielectric constant, f=10 GHz is the center frequency, and $c=3\times 10^{8}$ m/s is light speed.

**Figure 3:**
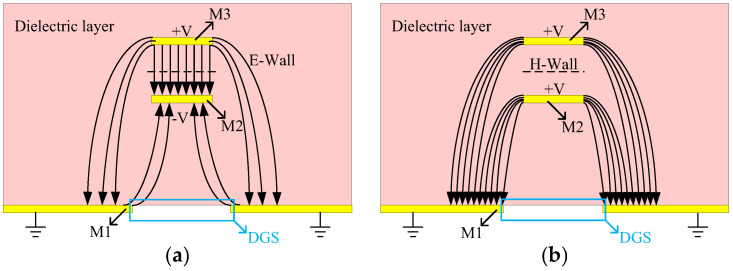
Electric field distribution diagram of coupled lines based on Defective Ground Structure (DGS): (**a**) ddd mode. (**b**) even mode.

The DGS structure is capable of addressing the weak coupling issue of the IPD coupler by providing band resistance, high characteristic impedance, and slow wave characteristics, as demonstrated in previous studies [24,25]. The characteristics of the DGS can be analyzed using odd- and even-modes analysis. Figure 3 shows the odd- and even-modes equivalent models for the proposed type A design in a side view. It can be observed that the odd-mode propagation has a relatively low current density on the ground plane, which allows for the neglect of the influence of odd-mode caused by DGS [26]. Due to the slow wave characteristic of the DGS, the even-mode velocity is reduced, while the odd-mode velocity remains almost constant. Consequently, from (2) and (3), the difference between the odd- and even-modes impedance becomes larger, thereby enhancing the coupling [27].

(2) $$Z_{0e}=\frac{1}{v_{pe}c}$$

(3) $$Z_{0o}=\frac{1}{v_{po}c}$$

**Figure 4:**
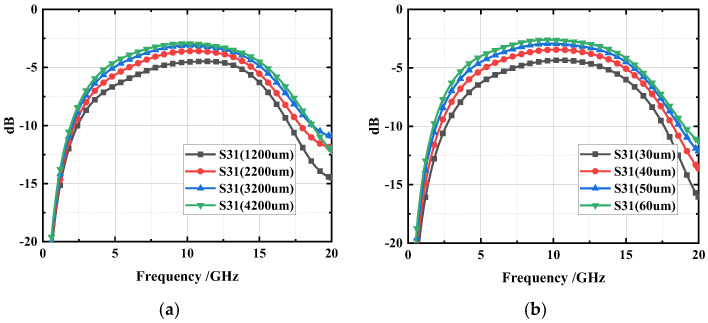
Influence of DGS structure on coupling of coupler: (**a**) length. (**b**) width.

**Figure 5:**
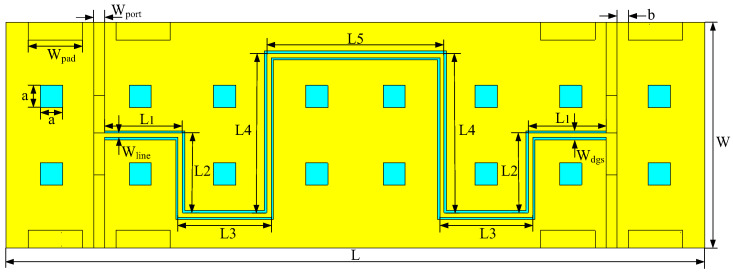
Top view of the proposed type A coupler.

To verify the characteristics of the DGS structure for coupling in the proposed coupler, simulations were conducted using Computer Simulation Technology (CST) software, with different geometric parameters for the DGS structure. The results are shown in Figure 4. It can be observed that the coupling is primarily determined by the length and width of the DGS structure. Keeping the width of the DGS structure constant and varying its length, the coupler was simulated at different lengths of the DGS, which were 4200, 3200, 2200, and 1200 µm, respectively. As shown in Figure 4a, the coupling of the coupler becomes tighter as the value of the length increases. Conversely, when the length is kept constant and the width is varied at 60, 50, 40, and 30 µm, respectively, the coupling becomes tighter as the value of the width increases, as shown in Figure 4b. These results can be attributed to the fact that a larger DGS area results in a greater difference between the odd- and even-mode phase velocities, leading to enhanced coupling. Therefore, the coupling can be flexibly adjusted by changing the size of the DGS structure. After simulation optimization, the length of the DGS structure was selected to be 4200 µm, and the width was chosen as 40 µm to achieve the desired coupling degree of 3 dB. The top view and dimensions of the type A design is shown in Figure 5, and the geometric parameters settings are shown in Table 1.

**Figure 6:**
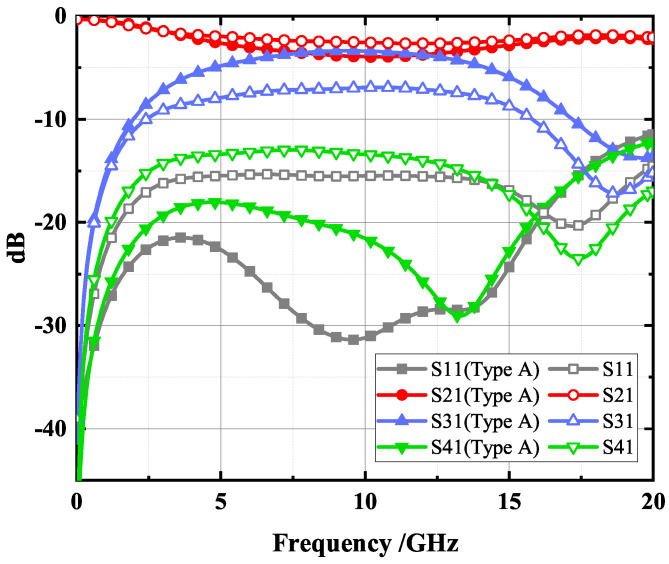
S—parameters simulation results for the structure with DGS and without DGS.

**Figure 7:**
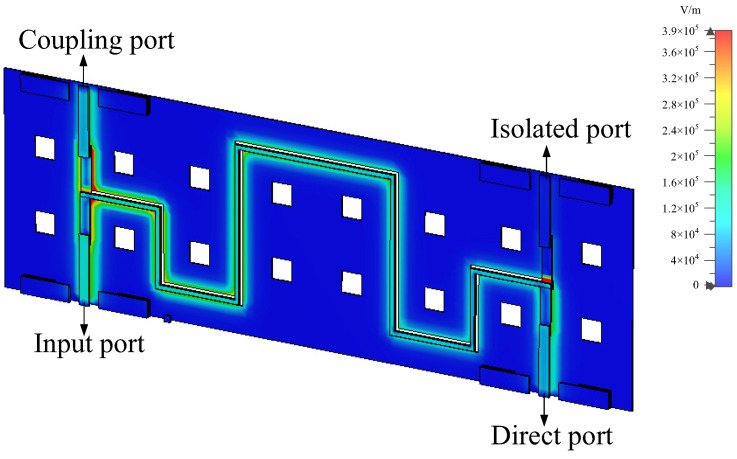
Electromagnetic (EM) simulation of the proposed type A coupler.

**Figure 8:**
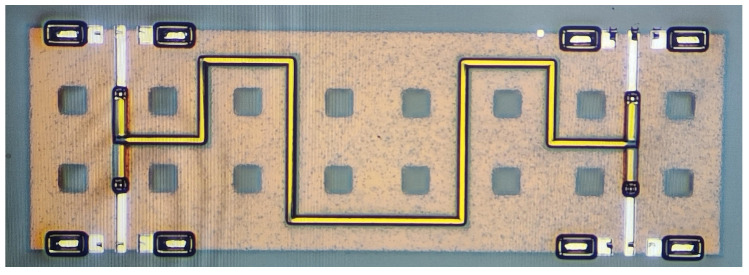
The chip layout photo of the proposed type A coupler.

The design was modeled and simulated in CST software, using a time-domain simulation solver with the simulation frequency set to 0–20 GHz. To better visualize the energy propagation, electric and magnetic field monitors at different frequencies were added in the simulation. Figure 6 provides a comparison of S-parameter simulation results for the structure with DGS and without DGS. The simulation results without the DGS, represented by the dotted lines, show a coupling degree of only about −7 dB, indicating poor isolation and return loss. The simulation results for the proposed type A coupler with a DGS structure, represented by the solid lines, show a coupling degree of −3.36 dB at the center frequency of 10 GHz and isolation and return loss of <−18.9 dB and <−28.5 dB, respectively, within the frequency range of 6.7–12.5 GHz. This effectively enhances the coupling degree and directivity of the coupler. Figure 7 shows the electromagnetic (EM) simulation result of the proposed type A coupler at 10 GHz, where the power input to coupler can be divided into direct and coupling ports, while isolation port has no power output. Based on the above EM simulation results, the proposed type A coupler was fabricated on the IPD process, and the chip layout photo is shown in Figure 8.

### 2.3. Type B IPD Coupler

**Figure 9:**
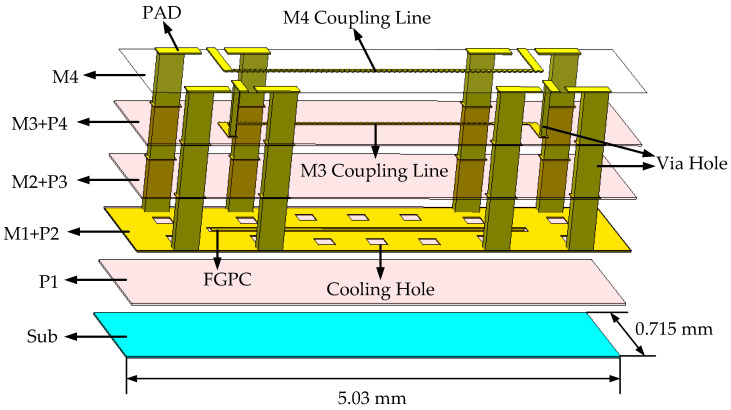
A 3D view of the type B IPD coupler.

The three-dimensional (3D) view of the proposed type B coupler is shown in Figure 9. This design uses M4 and M3 metal layers for broadside-coupling to minimize conductor loss and improve the quality factor of the passive device. Different from type A, type B adopts a floating ground-plane conductor (FGPC) structure to enhance the coupling and further improve the directivity of the coupler. Additionally, the coupling lines are fine-tuned with a swing structure to match the odd- and even-modes speeds for better directivity. This design provides good isolation and return loss not only at 10 GHz, but also in the next two frequency bands. Similar to type A, the via holes are used to connect the signals to the top layer, the cooling holes are required for machining, and four ground–signal–ground (GSG) pads are matched to 50 Ω.The size of the proposed type B coupler is 5.03 mm × 0.715 mm.

**Figure 10:**
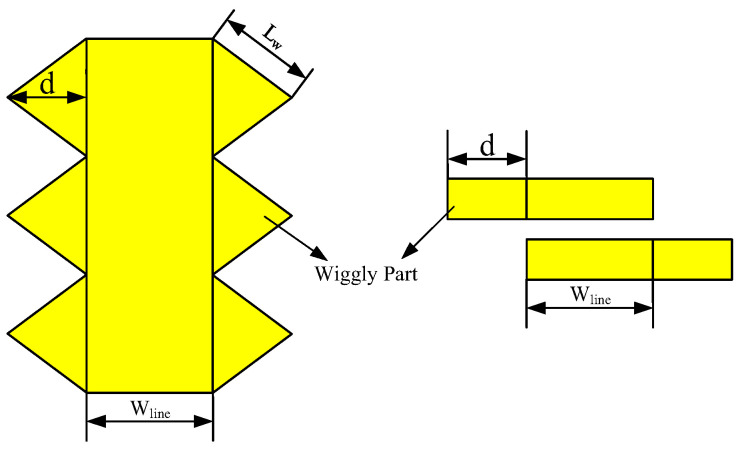
Top view and side view of the wiggly-coupled line.

**Figure 11:**
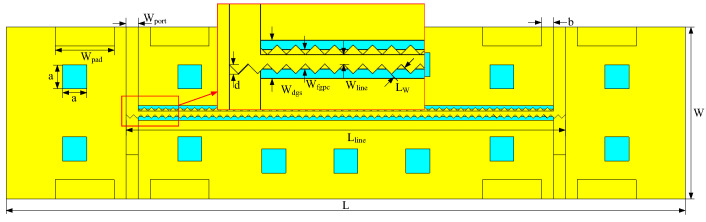
Top view of the proposed type B coupler.

The wiggly lines with edge-coupling can effectively improve the directivity, and has been confirmed in [28,29]. When the coupled line is in a nonuniform medium, the directivity of the coupler can be poor due to the uneven phase velocities of its odd- and even-modes. To address this issue, the type B coupler proposes wiggly lines with broadside-coupling that decelerate the odd-mode phase velocity without affecting the even-mode phase velocity. This leads to an improvement in the directivity of the directional coupler. The proposed wiggly lines are depicted in Figure 10, where the top and side views are shown on the left and right, respectively. In this design, $L_{w}$ represents the side length of the triangle, *d* denotes the height of the triangle, and *w* represents the line width. Similar to edge-coupled wiggly lines [30], if $C_{o}$ is used to represent the unit equivalent odd-mode capacitance of the straight-coupled line, the unit equivalent capacitance of the wiggly-coupled line can be represented by $C_{ow}$ as

(4) $$C_{ow}=C_{o}+C_{w}$$

where $C_{w}$ is the odd-mode capacitance affected by the wiggly lines. The odd-mode effective permittivity $\varepsilon_{reow}$ and odd-mode phase velocity $v_{pow}$ are, respectively, represented by

(5) $$\varepsilon_{reow}=\frac{C_{ow}}{C_{o0}}$$

(6) $$v_{pow}=\frac{c}{\varepsilon_{reow}}$$

where $C_{o0}$ is the odd-mode capacitance under the condition that the material is replaced with a unit dielectric constant material and *c* is the speed of light. Therefore, with the implementation of wiggly-coupled lines, the odd-mode capacitance increases, resulting in an increase in the relative dielectric constant of the odd-mode. This causes the odd-mode speed to slow down and match the speed of the even-mode. Finally, after simulation optimization, the top view and dimensions of the type B coupler is shown in Figure 11, and the geometric parameters settings are presented in Table 2.

**Figure 12:**
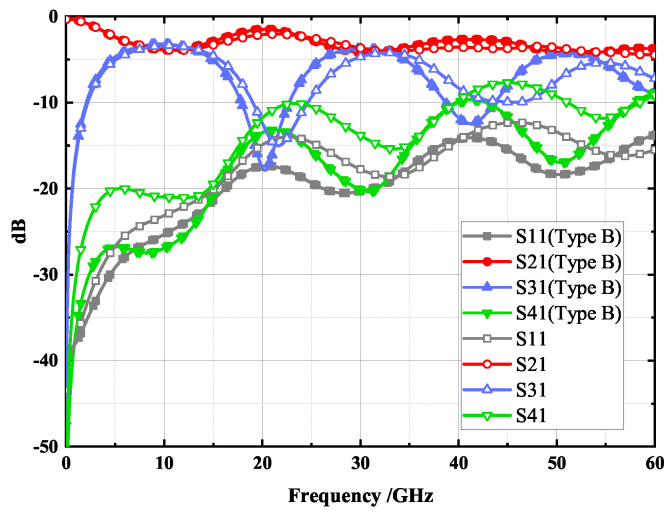
S—parameters simulation results with a swing coupling line and straight coupling line.

**Figure 13:**
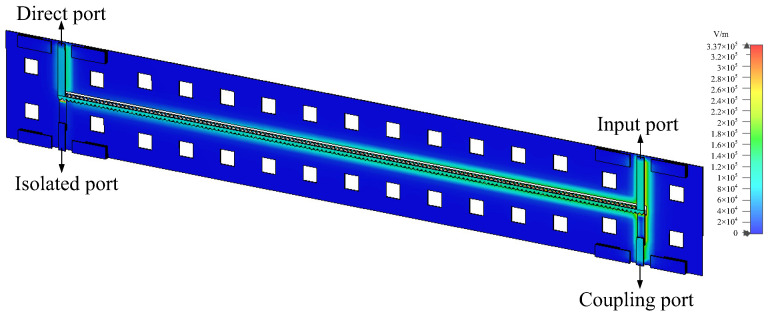
EM simulation of the proposed type B coupler.

**Figure 14:**
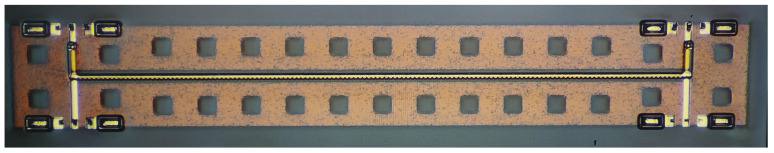
The chip layout photo of the proposed type B coupler.

As with type A, modeling and the simulation were conducted in CST software with similar parameters set, except for the frequency range, which was extended to 0–60 GHz. The simulation results of the proposed type B coupler are shown in Figure 12, where the solid lines are the simulation results of the wiggly-coupled lines, while the dotted lines are the simulation results of the straight-coupled lines. It is evident that the coupler with the wiggly-coupled lines exhibits better return loss and isolation, thus effectively improving directivity, especially in the latter two frequency bands. Figure 13 illustrates the EM simulation result of type B coupler at 10 GHz. As shown, the power input to the coupler can be divided into direct and coupling ports, while the isolation port has no power output, achieving 3 dB coupling. According to these results, the proposed type B coupler was manufactured using the IPD process, and a microphotograph of the chip layout is shown in Figure 14.

## 3. Measurement and Discussion

### 3.1. Measurement System

**Figure 15:**
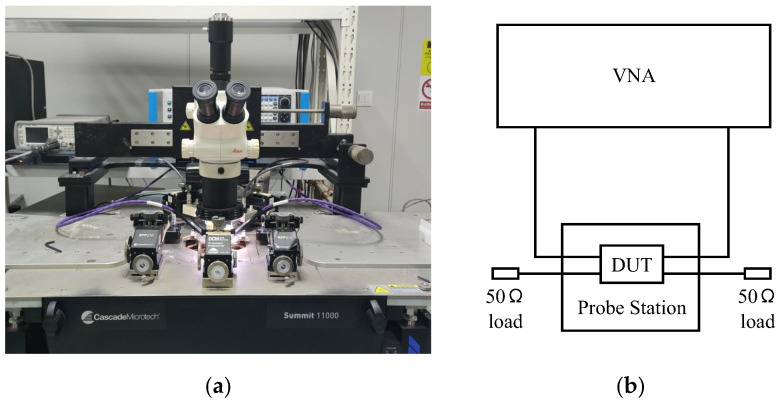
(**a**) Measurement system of the coupler. (**b**) Measurement system topology graph.

The performance of the two types of couplers was measured using a Vector Network Analyzer (VNA), as shown in Figure 15a. The measurement system topology graph of the coupler is shown in Figure 15b. A VNA (Ceyear 3672E) with a frequency range of 10 MHz to 67 GHz was used to measure the S-parameter of the coupler. The device under test (DUT) was placed on the probe station (Cascode Summit 11000). A short-open-load-thru (SOLT) calibration was performed on the on-wafer. In order to measure the full characteristics of the fabricated couplers using a two-port VNA, three measurements were carried out with different two-port terminations (50 Ω load).

### 3.2. Measurement Results

#### 3.2.1. Type A IPD Coupler

**Figure 16:**
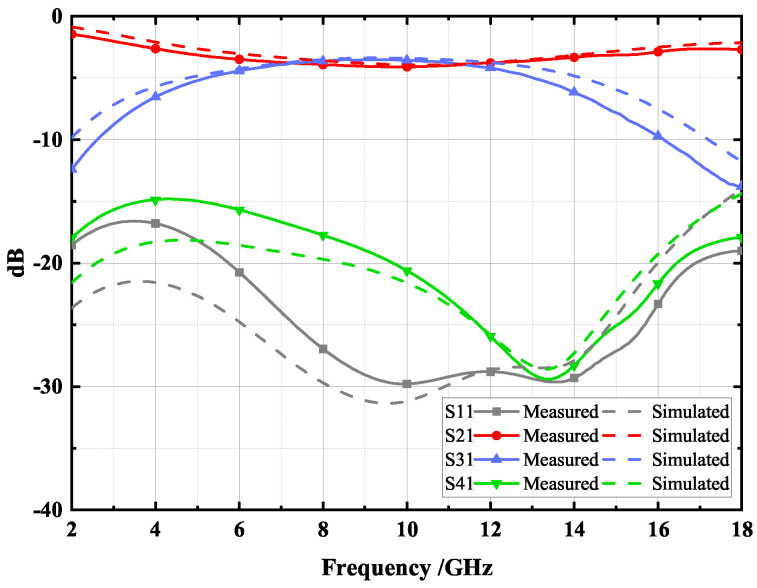
Simulation and measurement results of the proposed type A coupler.

The measured S-parameters results of the proposed type A coupler are shown in Figure 16 and are consistent with the simulated results. The measurement results demonstrate that the proposed type A coupler at the center frequency of 10 GHz exhibits a 3 dB coupling response at 6.5–12.2 GHz, and a maximum coupling of 3.5 dB is obtained at 9.5 GHz. The proposed type B coupler has an isolation of less than 16 dB and return loss of less than 22 dB over a relative bandwidth of 60.96%. This good performance is attributed to the use of the DGS structure, which effectively enhances the coupling and improves the directivity of the coupler.

#### 3.2.2. Type B IPD Coupler

**Figure 17:**
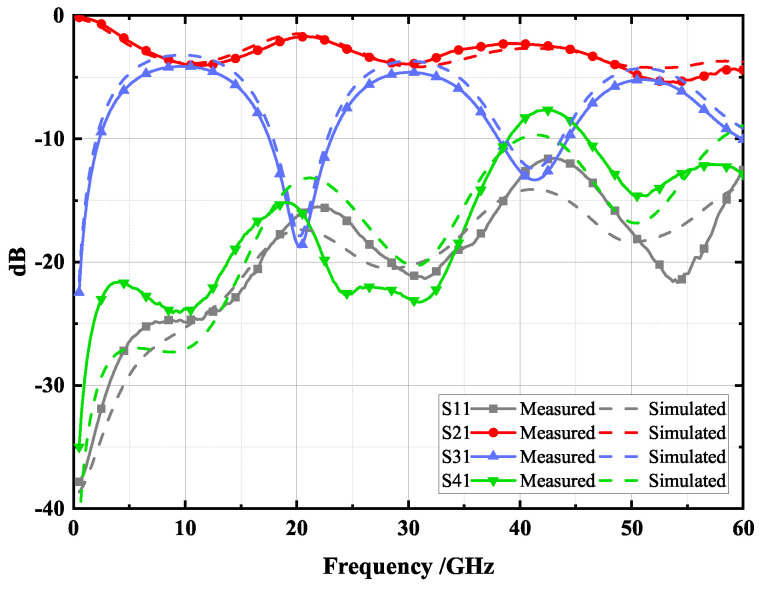
Simulation and measurement results of the proposed type B coupler.

Figure 17 illustrates the measured and simulated results of the proposed type B coupler, where the solid lines represent the measured results and the dotted lines represent the simulated results. As can be seen from the figure, the measured curves are in general agreement with the simulation curves. The strongest coupling in the first, second, and third bands are 4.13 dB, 4.61 dB, and 5.21 dB, respectively. The proposed type B coupler has <−21 dB isolation and <−23 dB return loss in the first band at 7–13 GHz; in the second band at 28–32.5 GHz, it has <−22 dB isolation and <−19 dB return loss; in the third band at 49.5–54.5 GHz, it has <−12 dB isolation and <−17 dB return loss. This performance enables the coupler to operate in three frequency bands, laying the foundation for a high efficiency harmonic controlled Radio Frequency Power Amplifier (RFPA) design.

### 3.3. Discussion

Table 3 compares the proposed 3 dB directional coupler with other designs of couplers in the previous literature. In general, couplers designed based on the LTCC process exhibit better performance but have a lower operating frequency, narrower bandwidth, and larger physical size compared to the IPD couplers. The type A coupler designed in this paper has a better coupling ratio, return loss, isolation, and higher directivity compared to other IPD couplers, which is due to the adoption of the DGS structure. Additionally, the type B coupler, which adopts wiggly coupling lines, can operate in three frequency bands without the sacrificing coupling ratio and return loss, providing a new idea for increasing the coupler bandwidth. This is a great advantage, considering that normally, multiple couplers must be cascaded to adapt the design of triple-band PA, which not only meets the linearity requirements of various wireless communication protocols in a single RF front-end, but also reduces the size of the integrated circuit. As to the best knowledge of the author, there is no previous research reported of such couplers using the HI process.

## 4. Conclusions

In this paper, two innovative on-chip 3 dB directional couplers are proposed using the IPD process. To overcome the weak coupling ratio in the IPD multilayered substrate, the type A coupler adopts the DGS structure to switch the phase velocity of odd-even modes, resulting in a significant enhancement of the coupling. The type B coupler utilizes wiggly-coupled lines to match the phase velocity of odd- and even-modes, which makes the coupler have good isolation and return loss in three frequency bands, thus improving directivity. As demonstrated in Table 3, both proposed couplers exhibit a wide bandwidth and would be highly advantageous in the design and integration of multi-band RF front-end.

## Figures and Tables

**Table 1:** Geometric parameters of proposed type A coupler.

*L*	L1	L2	L3	L4	L5	*W*	$W_{\mathrm{line}}$	$W_{\mathrm{dgs}}$	$W_{\mathrm{port}}$	$W_{\mathrm{pad}}$	*a*	*b*
3.15 mm	0.35 mm	0.36 mm	0.42 mm	0.72 mm	0.8 mm	1.02 mm	20 µm	40 µm	50 µm	245 µm	100 µm	50 µm

**Table 2:** Geometric parameters of the proposed type B coupler.

*L*	$L_{\mathrm{line}}$	$L_{w}$	*W*	$W_{\mathrm{line}}$	$W_{\mathrm{dgs}}$	$W_{\mathrm{fgpc}}$	*d*	$W_{\mathrm{port}}$	$W_{\mathrm{pad}}$	*a*	*b*
5.03 mm	4.23 mm	$15\sqrt{2}$ mm	0.715 mm	15 µm	60 µm	30 µm	15 µm	50 µm	245 µm	100 µm	50 µm

**Table 3:** Performance summary of the coupled-line couplers.

Ref.	Frequency Range (GHz)	FBW(%)	Max. Coupling (dB)	Return Loss (dB)	Isolation (dB)	Circuit Size	Fabrication Process
[31]	1.5–2.2	19	3.3	<−27	<−35	16.2 mm${}^{2}$ $0.003{\lambda_{0}}^{2}$	LTCC
[32]	2.65–3.55	29.03	3.25	<−24	<−26	21.6 mm${}^{2}$ $0.00231{\lambda_{0}}^{2}$	LTCC
[18]	3.6–5	32.5	4.2	<−14	<−15	0.69 mm${}^{2}$ $0.0001{\lambda_{0}}^{2}$	IPD
[15] Type-A	33.3–53.9	47.2	4.67	<−15	<−18.5	0.4994 mm${}^{2}$ $0.00078{\lambda_{0}}^{2}$	IPD
[16]	4.8–6.2	25.45	N.A.	<−17	<−17	0.7954 mm${}^{2}$	IPD
[33]	57–64	10.37	4.9	<−15	<−17	0.4383 mm${}^{2}$ $0.01753{\lambda_{0}}^{2}$	IPD
This work Type-A	6.5–12.2	60.96	3.5	<−22.32	<−16.16	3.213 mm${}^{2}$ $0.01143{\lambda_{0}}^{2}$	IPD
This work Type-B	7–13	60	4.13	<−23.95	<−21.21	3.5965 mm${}^{2}$ $0.01279{\lambda_{0}}^{2}$	IPD
This work Type-B	28–32.5	14.88	4.61	<−19.67	<−22.17	3.5965 mm${}^{2}$ $0.01279{\lambda_{0}}^{2}$	IPD
This work Type-B	49.5–54.5	8.78	5.21	<−17.02	<−12.79	3.5965 mm${}^{2}$ $0.01279{\lambda_{0}}^{2}$	IPD

## References

[1] Li C., Zhang F., Di M., Pan Z., Wang A. Advances in 3D Heterogeneous Structures and Integration for Future ICs (Invited). Proceedings of the 2019 IEEE SOI-3D-Subthreshold Microelectronics Technology Unified Conference (S3S).

[2] Gutierrez-Aitken A. Heterogeneous Integration for High Frequency RF Applications. Proceedings of the 2020 IEEE International Electron Devices Meeting (IEDM).

[3] Zhou J., Yang J., Shen Y. 3D heterogeneous integration technology using hot via MMIC and silicon interposer with millimeter wave application. Proceedings of the 2017 IEEE MTT-S International Microwave Symposium (IMS).

[4] Xinhai B., Hongyan G., Li Z., Tan K.H., Lai C.M. Fabrication and measurement of BPF using IPD technology. Proceedings of the 2014 15th International Conference on Electronic Packaging Technology.

[5] Lee P.N., Hsieh Y.C., Hsieh S.C., Kung C.Y., Wang C.C. Design and Fabrication of Band-Pass Filter on Glass IPD for 5G New Radio. Proceedings of the 2020 IEEE 70th Electronic Components and Technology Conference (ECTC).

[6] Chen H.K., Hsu Y.C., Lin T.Y., Chang D.C., Juang Y.Z., Lu S.S. CMOS wideband LNA design using integrated passive device. Proceedings of the 2009 IEEE MTT-S International Microwave Symposium Digest.

[7] Jeon H., Park Y., Huang Y.Y., Kim J., Lee K.S., Lee C.H., Kenney J.S. (2012). A Triple-Mode Balanced Linear CMOS Power Amplifier Using a Switched-Quadrature Coupler. IEEE J. Solid-State Circuits.

[8] Lu H.C., Kuo C.C., Wei S.A., Huang P.S., Wang H. Ultra broad band CMOS balanced amplifiers using quadrature power splitters on glass integrated passive device (GIPD) and LTCC with flip chip interconnects for SiP integration. Proceedings of the 2012 IEEE/MTT-S International Microwave Symposium Digest.

[9] Hsu Y.C., Chiou H.K., Chen H.K., Lin T.Y., Chang D.C., Juang Y.Z. (2011). Low Phase Noise and Low Power Consumption VCOs Using CMOS and IPD Technologies. IEEE Trans. Components Packag. Manuf. Technol..

[10] Wei M.D., Chang S.F., Negra R. Design of low phase noise K-band Voltage-Controlled Oscillator using 180 nm CMOS and integrated passive device technologies. Proceedings of the 2014 NORCHIP.

[11] Kim H.T., Liu K., Frye R.C., Lee Y.T., Kim G., Ahn B. Design of compact power divider using integrated passive device (IPD) technology. Proceedings of the 2009 59th Electronic Components and Technology Conference.

[12] Hsiao C.Y., Hsu S.S.H., Chang D.C. (2011). A Compact V-Band Bandpass Filter in IPD Technology. IEEE Microw. Wirel. Components Lett..

[13] Pan D., You B., Wen X., Li X. (2022). Wideband substrate integrated waveguide chip filter using spoof surface plasmon polariton. Micromachines.

[14] Chen Y.T., Chang C.L., Tseng C.H. A compact X-band CPW branch-line coupler using glass integrated passive device (GIPD) technology. Proceedings of the 2013 Asia-Pacific Microwave Conference Proceedings (APMC).

[15] Tseng C.H., Chen Y.T. (2016). Design and Implementation of New 3-dB Quadrature Couplers Using PCB and Silicon-Based IPD Technologies. IEEE Trans. Components Packag. Manuf. Technol..

[16] Jeon H., Kobayashi K.W. (2018). Comparison of 5-GHz Quadrature Couplers Using GaAs and Silicon-Based IPD Technologies. IEEE Microw. Wirel. Components Lett..

[17] Shim S., Hong S. (2011). A CMOS Power Amplifier With Integrated-Passive-Device Spiral-Shaped Directional Coupler for Mobile UHF RFID Reader. IEEE Trans. Microw. Theory Tech..

[18] Tseng Y.C., Ma T.G. On-chip miniaturized 3-dB directional coupler using coupled synthesized CPWs on integrated passive device (IPD) process. Proceedings of the 2014 44th European Microwave Conference.

[19] Lin K.C., Chiou H.K., Chien K.H., Yang T.Y., Wu P.C., Ko C.L., Juang Y.Z. (2013). A 4.2-mW 6-dB Gain 5–65-GHz Gate-Pumped Down-Conversion Mixer Using Darlington Cell for 60-GHz CMOS Receiver. IEEE Trans. Microw. Theory Tech..

[20] Alizadeh A., Frounchi M., Medi A. (2016). On Design of Wideband Compact-Size Ka/Q-Band High-Power Amplifiers. IEEE Trans. Microw. Theory Tech..

[21] Brown M., Li C. A K-Band Broadband Binary Phase Shifter. Proceedings of the 2022 IEEE Radio and Wireless Symposium (RWS).

[22] Lin Y.S., Lee J.H. (2013). Miniature Butler Matrix Design Using Glass-Based Thin-Film Integrated Passive Device Technology for 2.5-GHz Applications. IEEE Trans. Microw. Theory Tech..

[23] Wang Q., Guo Y. Design and Implementation of High Precision and High Density Capacitor based on Silicon Integrated Passive Device Technology. Proceedings of the 2022 23rd International Conference on Electronic Packaging Technology (ICEPT).

[24] Liang C.H., Chang W.S., Chang C.Y. (2011). Enhanced Coupling Structures for Tight Couplers and Wideband Filters. IEEE Trans. Microw. Theory Tech..

[25] Velazquez-Ahumada M., Martel J., Medina F. (2004). Parallel coupled microstrip filters with ground-plane aperture for spurious band suppression and enhanced coupling. IEEE Trans. Microw. Theory Tech..

[26] Hsu S.K., Lin T.H., Wu T.L. Size-reduction of 3 dB microstrip forward-wave coupler using defected ground structure. Proceedings of the Asia-Pacific Microwave Conference 2011.

[27] Pozar D.M. (2011). Microwave Engineering.

[28] Uysal S., Aghvami H. (1989). Synthesis, design, and construction of ultra-wide-band nonuniform quadrature directional couplers in inhomogeneous media. IEEE Trans. Microw. Theory Tech..

[29] Sorkin O., Holdengreber E., Averbukh M., Schacham S.E., Farber E. Directivity Enhancement of Tight Couplers. Proceedings of the 2019 IEEE International Conference on Microwaves, Antennas, Communications and Electronic Systems (COMCAS).

[30] Bahl I., Mongia P.B.R. (2001). RF and microwave coupled-line circuits. Microw. J..

[31] Sawicki A., Sachse K. (2003). Novel coupled-line conductor-backed coplanar and microstrip directional couplers for PCB and LTCC applications. IEEE Trans. Microw. Theory Tech..

[32] Sun C., Dai Y., Li B., Li Y. Design of wide-brimmed coupler based on LTCC technology. Proceedings of the 2016 IEEE International Conference on Microwave and Millimeter Wave Technology (ICMMT).

[33] Haroun I., Plett C., Hsu Y.C., Chang D.C. (2012). Compact 60-GHz IPD-Based Branch-Line Coupler for System-on-Package V-Band Radios. IEEE Trans. Components Packag. Manuf. Technol..

